# Electricity as a Cause of Disease

**Published:** 1854-07

**Authors:** S. Littell

**Affiliations:** Surgeon to Wills Hospital for Diseases of the Eye; Philadelphia


### MEDICAL EXAMINER.

NEW SERIES.—NO. CXV.—JULY, 1854.

### ORIGINAL COMMUNICATIONS.

*Electricity as a Cause of Disease.* By S. LITTELL, M. D., Surgeon to Wills Hospital for Diseases of the Eye.

In no department of medicine is there more crude and unfounded theory than that which treats of the etiology of disease. The opinions prevalent on this subject are, indeed, hardly creditable to us as members of a learned profession, because they prove that we have not been guided in our reasoning by sound principles of philosophy. Simplicity is found to be an attribute of the ALMIGHTY in all the operations of His hand; we are amazed at the variety and diversity of the results produced by the combinations of a few simple elements; and have reason to believe that, as our knowledge increases, this characteristic will be still more apparent. Why should not the same be true also of the animal economy? It is a complex and intricate organism, subjected to the control of a central power—the brain—from any change, in the action of which, innumerable deviations from a normal condition might, a priori, be anticipated. How much more philosophical, then, to recognize a single principle capable of producing such change, than needlessly to multiply causes, and invoke the interposition of as many agencies as there are diseases in the nosology! We have imaginary miasms for the several exanthemata, for influenza, for cholera, for each of many different kinds of fever, for hooping cough, mumps, &c. &c. In accounting for the phlegmasiæ, it is true, we are contented to veil our ignorance and flatter our vanity under the comprehensive phrase of "taking cold;" an expression, however, to which we attach no definite ideas, and which, in its literal sense, is proved to be incorrect by circumstances of daily occurrence.

An etiology so manifold cannot be true; and if the abnormal manifestations may, in very many cases, be more satisfactorily explained through the instrumentality of a single principle, it must be abandoned.

The analogy existing between the nervous force and electricity has long been known; their actual identity has been rendered not improbable; and with this hint to guide inquiry, it is surprising that observation and reflection have not led to more important results. The arguments adduced by some physiologists to disprove this identity, and establish instead a state of "correlation," are far from being conclusive; and even if this were admitted, it would not militate against the view that I propose, which only requires that the nervous force should be affected by the mutations of the electrical fluid. Under the influence of vitality there would necessarily be some resistance to external agencies, and deductions drawn from experiments performed upon inanimate portions of a nerve, or upon the living nerve in an unnatural condition, are open to obvious objection. Light heat and electricity are now regarded as probable modifications of the same element, and there is no reason why, under another modification, that element should not constitute the vis nervosa also. A strong presumption of their substantial sameness, notwithstanding the reasoning which would invalidate it, is afforded by the existence of several species of fish with electrical organs; the action of which is dependent upon their connection with nervous centres, varies in intensity with the extent of that connection and the health of the animal, is under the control of the will, and, through a continued series of discharges, is capable of exhausting the nervous energy in a degree sufficient, in some cases, to occasion death.

I regard the brain and nervous system as an apparatus through the medium of which, electricity—the great agent of PROVIDENCE in the production of most of the phenomena of nature—modified and restrained by certain laws, is made subservient to the purposes of life; in other words, as a vital electrical machine through which that subtle fluid is both separated and distributed in accordance with the wants of the economy. If this hypothesis be correct we should naturally expect that, though to a great extent independent of external influences, it would yet be liable to be affected by fluctuations in the quantity of that fluid in the surrounding elements; and that this disturbance, moreover, would be more considerable while the organ, mutable and unconfirmed in its action, was in a state of greater proportional development, as in childhood. What we should thus anticipate, has been shown to be true in fact; electricity, when present in excess, exciting the functions and exalting vitality, while the contrary effect is produced by its deficiency. In a state of health and mature existence, when, all the functions being vigorously performed, the resisting power is greatest, the disturbing influence of such variations is comparatively slight, but under other circumstances they become a frequent and potential cause of disease. We have all experienced the feeling of energy and elasticity which is imparted when the electrical fluid is present in due proportion in the atmosphere, as in clear cold weather, and more strongly still the sensations of chilliness and discomfort occasioned by its deficiency under opposite conditions. Rheumatic persons, and those who have recently suffered from sprained or fractured limbs, can predict with unerring certainty an approaching atmospherical change, though to others there may be no sensible indications of its occurrence; and the evening exacerbations, which we observe in fevers and other complaints, are owing to the same cause,—the system, in its disturbed or debilitated condition, being unable to bear, without suffering, electrical changes which would exert little or no perceptible effect in a healthy state. It is these changes, moreover, consequent upon the withdrawal of the sun's rays, precipitating the dew, and thereby increasing the conducting power of the air, which render exposure at this time so dangerous in certain seasons, and not, as has been supposed, the greater prevalence on such occasions of miasmatic exhalations. The extreme sensibility to such impressions of the affections which are purely nervous, is a subject of common observation. Every physician must have noticed the great frequency of asthmatic attacks before a change of weather. The epileptic paroxysm occurs also most frequently in the night; and while this may perhaps be explained in part by the temporary suspension of the will in sleep, it is not irrational to attribute it in some degree to electrical changes, which are both more common and more prejudicial at that period; especially as we know that some persons, subject to this malady, are affected only at the vernal and autumnal equinoxes, when these fluctuations are greater than other times.*

The familiar expression of "taking cold," which is supposed to account so satisfactorily for many of the ailments to which flesh is heir, may be mentioned as another example of such influence. This is owing, not to variations of temperature, as generally believed, but to disturbances in the electrical condition of the atmosphere, of which these variations are effects or accompaniments. It is not unusual for individuals, especially those of tender age, to retire in apparent health to rest, in a comfortable room and bed, and to awaken after some hours with a sore throat, or a paroxysm of croup; and we have all known persons to be attacked with these and other complaints said to arise from "cold," who have been closely confined for days or weeks to apartments, the air of which has been steadily maintained at an elevated temperature. They could only have been affected therefore by changes in the electrical constitution of the air without, and these, though less likely to be injurious under such circumstances, would be felt with the instantaneousness of thought, however situated. It is worthy of remark that these effects are chiefly produced in the elemental changes which precede a storm, and less frequently during its continuance; when the rain or snow begins to fall, the electrical equilibrium is restored, and, if the vascular system has not become involved, neuralgic pains and

* Rheumatism scarcely excepted, there is not a disease in the whole catalogue, the phenomena of which are more in harmony with the electrical theory than those of epilepsy.

uncomfortable sensations subside. The knowledge of this fact has induced me, where I have the privilege of choice, to postpone important and delicate operations—those on the eye, for example—when a change of weather is impending; preferring rather to operate while the rain is actually falling, than during the dispositions preliminary thereto.

The effect on the gravid female of certain atmospherical conditions has been long observed. "Cold, rainy weather, and low damp miasmatic localities," says Professor Gilman, "have been recognized since the days of Hippocrates, as disturbing pregnancy and causing abortion. To the influence of the atmosphere is to be attributed the frequency of abortion, miscarriage, or other mishap in pregnancy by which some years are signalized." The probable explanation is, that the expenditure of nervous energy in the reproductive process, renders the system more liable to be affected by such changes. Insane persons, on the other hand, have always been, in a remarkable degree, insensible to atmospherical vicissitudes as well as exempt from epidemical impressions, and this exemption is owing to the habitual exaltation of cerebral action in their case. It is stated that not one inmate of the Lunatic Asylum died during the pestilence which lately desolated New Orleans.

These, and a host of similar facts, may be adduced to prove that there is nothing improbable in the hypothesis, that under circumstances of predisposing or concomitant influence, general or individual, pathological effects of far greater gravity, variety and extent, may be occasioned by the exhaustion of nervous energy through changes in the distribution of the electrical fluid; and that the exhaustion thus induced, may be the proximate cause not only of the exanthemata and most other forms of fever, congestive and otherwise, but also of cholera, influenza, hooping cough, erysipelas, the idiopathic phlegmasiæ, &c., &c.

The long continuance, in various degrees and combinations, of heat, drought, cold, and humidity, or the marked predominance of any one of these conditions, will create predispositions which determine the general character of the prevailing diseases. A hot and dry summer will be nosologically distinguished by affections of the alimentary canal, and will be followed by fevers of various type and degrees of severity; a peculiarly raw and chilly atmosphere precedes and accompanies influenza, a disease which is often characterized by excessive disturbance of the nervous system; while a cold, wet, and variable state of weather is favorable to the production of the exanthemata, typhoid fever, erysipelas, &c. Among the circumstances alluded to as affecting individuals, and thereby giving efficiency to electrical vicissitudes, may be enumerated fatigue, fasting, loss of sleep, exposure to a chilly atmosphere, the depressing passions, and whatever tends to debilitate the frame and exhaust or diminish nervous power.

The indirect action of heat during the summer months excites the abdominal viscera into unnatural activity, and gives rise to diarrhœa, dysentery, cholera morbus, &c. Debility ensues as a consequence of its prolonged operation, and in ordinary seasons the same predisposition continuing in a mitigated form, intermittent and remittent fevers—in which the vascular change is rather congestive than inflammatory—chiefly prevail during the early autumnal months. A higher grade of heat, producing a greater degree of exhaustion, and increasing the inflammatory tendency, which expends itself principally on the stomach and bowels, is the cause of yellow fever. At a later period, the system re-acting under the invigorating influence of cold, the predisposition again changes, and, as a general rule, eruptive fevers and organic inflammations abound.

The view which I have taken of this subject does not rest wholly on hypothesis. The diminution of magnetic power during the prevalence of cholera, has been ascertained by actual experiment. Mr. Mather, of South Shields, England, states that in 1849, when cholera of a very fatal character was epidemic in his neighborhood, he found, as the result of numerous observations carefully made, that a magnet which ordinarily carried two pounds and ten ounces, would, when the atmospheric indications were nearly at their worst—the air being almost saturated with moisture—sustain only one pound and ten ounces; the degree of its attraction varying with, and being in inverse proportion to, the virulence of the disease. In the same year the number of deaths in Paris from this pestilence, rapidly increased until the eighth of June, when they amounted to six hundred and twenty-three. On the evening of that day there occurred a thunder-storm of unusual severity, and the cholera immediately began to decrease; by the eighteenth, there was a daily report of one hundred only, and at the close of the month, the mortality had fallen to thirty. Similar observations have been recorded by others; and from a consideration of all the circumstances attending this disease, its preference for the great water courses of a country, &c., its electrical origin may be regarded as pretty certainly established. The meteorological constitution in which influenza appears, would incline us to predict with certainty a like condition of things in reference to that complaint; and all the diseases above enumerated, are notoriously most frequent—indeed they prevail almost exclusively—in seasons when electrical changes are greatest, and their injurious operation aggravated by cold, moisture, and other depressing or auxiliary influences. There are many facts, moreover, irreconcilable with the commonly received notion of the malarious production of fever; and without absolutely denying the deleterious action on the animal economy of exhalations from decaying vegetable matter, I am fully convinced that a part far too prominent and exclusive has been attributed to their agency. The theory afforded a plausible solution of many things hard to be understood, and being supported by a multitude of apparent facts, has been too hastily received and adopted by the profession. The complaints supposed to be thus engendered, prevail at a period when electrical vicissitudes are greatest, and the body, debilitated and otherwise disordered by the protracted heat of summer, is most sensible to their impression; they are often observed where there is no reason to suspect the operation of malaria; are notoriously reproduced by other causes after they have once occurred; and are promptly cured by means which eliminate no poison, but merely restore the lost tone of the system—frequently, indeed, by mental impressions alone.

That such diversity of effect should be produced by the same morbific agent constitutes, as has been seen, no valid objection to the hypothesis which we advocate. Man in his ignorance is fond of multiplying causes, but science is daily demonstrating the simplicity of truth. In the present instance there is no necessity for a multifarious machinery. Defective innervation may give rise to aberrations as various as there are tissues and organs to be acted upon. Thus, in the dermoid tissue we may have derangement of the *capillary circulation*, of the *exhalants*, or of the *secretory apparatus*, constituting respectively, scarlatina, rubeola and small pox; or we may have cholera from the same cause directed to the mucous membrane of the stomach and bowels; or any one of the phlegmasiæ, according as the development of latent imperfection, or accidental causes may determine. To those, therefore, who consider the complicated organism of the human frame, it will not appear strange that results apparently so different, and yet in reality essentially the same, should be produced through the instrumentality of a single principle directed in its morbid manifestations by predispositions arising from a variety of circumstances, existing in countless combinations, and involving whole communities, or affecting individuals only.

Its modus operandi may be briefly explained, and will perhaps be best understood by a reference to scarlet fever. This disease, though occasionally observed in every season of the year, prevails most extensively from October to April or May, a period during which electrical fluctuations are greatest, and their influence promoted by the various circumstances already mentioned; and, as might be expected, is chiefly confined to children, whose power of resisting hurtful impressions is less than that of persons of mature age and vigor. The functions of the brain being dedepressed or otherwise disturbed by the rapid abstraction of sensorial energy, the control of that organ over the capillaries is lessened, they consequently become dilated, the circulation through them is retarded, and a condition is induced closely bordering on inflammation. This, though general in all the tissues, is more particularly observed in the mucous membrane of the digestive system and in the skin, as evinced in the former, by the redness of the throat and the projecting papillæ of the tongue, and in the latter, by the scarlet efflorescence and other symptoms of increased action; the predominance of which in this tissue generally indicates a tractable form of the complaint. Meanwhile, the brain reacting against the morbific agency separates and transmits the nervous energy as before; but there is now a demand for a greater supply, in order to secure the restoration of the impaired tonicity of the capillaries. This is accomplished through the exaltation of its functions produced by the febrile movement—an action of salutary tendency when it does not transcend the required limits—and after a commotion of greater or less severity, occupying necessarily a nearly definite period, the system reverts to a state of convalescence. Such is the order of things in scarlatina simplex. In the anginose variety the pathological alterations proceed one step further. The circulation through a portion of the capillaries is not only retarded but actually arrested, accumulation follows, and inflammation is set up in the fauces, where, from the laxity of the parts and the exposed condition of the vessels, we should naturally expect to find it. In still more aggravated grades of the malady, whether owing to the intensity of the cause, feebleness of constitution, or some other circumstances affecting the individual, the powers of life are prostrated, in many instances beyond the capability of reaction; the brain being deprived of its nervous energy coma sometimes ensues, innervation is suspended, congestion takes place in the larger organs, and after a struggle of varying duration death generally closes the scene—often supervening in very few hours.

The production of small pox and measles, when prevailing epidemically, or in sporadic cases where there has been no exposure to contagion, may be explained in like manner. The difference being, that in scarlatina the morbific influence exerts its force chiefly on the *capillary circulation;* whereas, in variola and measles, while it implicates—more particularly in the latter—the pulmonary, rather than the gastric, mucous tissue, it receives from some predisposing causes, a determination to different departments of the *secretory* apparatus of the skin, and from the elaboration which there takes place derives its specific and contagious character. In small pox it is not improbably the sebaceous follicles, and in rubeola the exhalants, which are thus affected.

The application of the same mode of reasoning to the phlegmasiæ is sufficiently obvious. An individual, from exposure in a raw and damp condition of the atmosphere—the physical powers being perhaps depressed by fasting, fatigue, or some other predisposing cause—becomes unwell, and is said in common parlance to have "taken cold." More correctly speaking, the sensorial energy has been abstracted from the system more rapidly than it could be generated without disturbance of the cerebral functions; the effect is felt in the diminished innervation of some organ liable, from congenital or acquired predisposition, to fall into diseased action; and as a consequence, inflammation takes place either in its parenchyma or investing membrane, as circumstances may determine.

The exanthemata prevail chiefly in early life, when the nervous system is not only predominant and impressible, but there is, moreover, from their greater functional activity, a natural tendency to affections of the dermoid and mucous tissues; in after years the predisposition inclines rather to disease of the contents of the great cavities. The exemption from a second attack, though by no means so general as is commonly supposed —especially in measles and scarlet fever—increases with the lapse of time, and, as might be expected, when they do recur, they are characterized by diminished severity; assuming, for the most part, the milder forms of scarlatina simplex, or rubeola sine catarrho. Various complaints originate in the same predisposition; and during the prevalence of these and other epidemics, there will be, of course, an increase of congeneric diseases. When the meteorological conditions favorable to the production of small pox exist, vaccination will confer little or no immunity.

I am far from supposing that fluctuations in the quantity of the element which I have mentioned, is the sole cause of morbid action. Disease once induced, has, in many instances, the power of self-propagation, and often originates, moreover, from other causes operating as well within as without the individual; but when it prevails epidemically, or in sporadic cases of complaints sometimes epidemic, where there has been no exposure to contagion, and on all occasions where it is said to arise from "cold," its etiology I believe to be as I have described.

Contagion itself, however, it may be remarked, acts very much in the same manner; not indeed by abstracting electricity from the brain, but by directly depressing the power of that organ, and thereby preventing it from emitting the due amount of nervous energy. The same series of consequences follow in the one case as in the other; thus, between typhus and typhoid fever the chief difference is in their causation, the train of morbid action being alike in both

When the predisposition is wanting, contagion will be nearly or altogether inoperative. Small pox itself under such circumstances will not spread beyond the individua laffected ; and may even give rise to affections of a different kind. I have seen cases of fatal congestive fever manifestly caused by attendance on the confluent variety of that disease.

The doctrine is fruitful in its practical applications, and involves whatever may protect the system against the fluctuations of this potent and all-pervading principle. It supplies us with an intelligible reason why in the selection of a residence we should reject localities the air of which is habitually charged with moisture and its conducting power thereby increased, whether from the vicinity of water or the character of the soil.* It would incline us, for a similar reason, to avoid exposure in feeble states of health to the early morning and evening air of the country, when intermittent and remittent fevers are rife, as in the fall; and when such exposure is unavoidable, points out the propriety of sustaining the vital powers by a previous meal and the exhibition of some tonic medicine. It explains how it happens that in certain seasons, and during the prevalence of certain winds, situations ordinarily salubrious become unhealthy.† It teaches us, moreover, during the existence of any epidemic to abstain from everything which may depress or exhaust the nervous energy, and to maintain the action of the brain in its acccustomed, or even in increased vigor, as well by the stimulus of hope and confidence, as by the use of means which exert their influence more especially upon that organ; affording thus a probable rationale of the operation of belladonna and other prophylactics. It guides us, furthermore, to a right practice in many affections now empirically treated, and indicates the true methodus medendi in any new phase which disease, originating as we have supposed, may hereafter assume ; we should, for example, refrain from venesection, and as much as possible from other debilitating

* Humidity alone, when *universal* and constant, tends to preserve the electrical equilibrium ; hence the salubrity of sea-air, and hence also the comparative healthfulness of a rainy autumn, so far at least as regards complaints of supposed miasmatic origin.

† The western side of rivers, that of the Schuylkill and Delaware, for instance, is more healthful than its opposite.

measures, in scarlet, typhoid, and other fevers; and in acute rheumatism, which involving in its commencement the nervous fibre itself is rapidly followed by the exhaustion of its excitability, we should endeavor to allay pain by anodynes, control vascular excitement by antimony, and early resort to means adapted to restore the impaired sensorial power. A want of due innervation being a primary deviation in the train of morbid action, it holds out a reasonable hope of subverting certain complaints in their incipient stage, by the employment of tonics, as quinine in large doses, before the vascular system has become actively implicated, and forbids a resort to the lancet at least until such implication has taken place, and reaction, permanently secured, threatens by its excess to endanger some important organ; this is a practice pursued in some parts of our country in pneumonia typhoides, it has been successfully adopted in influenza also, and reasoning from analogy should, with other appropriate remedies, be equally effective in the treatment of cholera.* It instructs us in some cases of chronic inflammation to restore and sustain the tone of the extreme vessels even while we endeavor to relieve local congestion by depletion—general invigoration and the topical abstraction of blood being not always incompatible. It abates in many instances the dread of contagion, and relieves us from the supposed necessity of purifying the blood by the elimination of an imaginary materies morbi; while by inculcating a sounder pathology it discountenances the common fallacy of inferring from the composition of perverted secretions, the remedies adapted to any particular complaint. It suggests the necessity of adequate clothing of appropriate quality—that is, of non-conducting material—and other precautionary measures in the management of children. And finally admonishes us of the importance at all times of preserving the vital forces in their best possible condition, and thereby of affording to the vis medicatrix naturæ full opportunity of accomplishing its recuperative tendencies.

It might, perhaps, be supposed on first impression, that disorders originating in a temporary abstraction of electricity, ought

* In children the exanthemata are frequently ushered in by convulsions or coma, and I have known the same thing to happen also in intermittent and other fevers; showing the primary action of the morbific cause upon the brain.

to be cured by the artificial supply of that fluid ; and this supposition would not be unreasonable if our bodies instead of being living systems were inanimate machines. In the actual constitution of things, however, other morbid actions speedily follow the temporary relaxation of cerebral control; various complications ensue; and effects are produced which can only be obviated or repaired in accordance with the laws which govern the animal economy alike in health and disease.

In what has been said, the substantial identity of the electrical fluid and the vis nervosa has been assumed; but, as before remarked, this is not necessary for the truth of the theory which I have advanced. It is sufficient that there should be such a reciprocal relation between them that fluctuations in the one will produce a corresponding change in the other—the morbid alterations being accounted for with equal clearness on either supposition,—and thus much, I presume, will be conceded by all physiologists.

The theme is a prolific one, and the general ideas thus briefly expressed, far from exhausting the subject, are intended merely as suggestive. They are not wholly original, for the electrical origin of several diseases has long been suspected, and as respects one of them, well nigh established ; but I am not aware that the hypothesis has ever received so extended an application. To my mind it harmonizes and explains many discordant and otherwise inexplicable phenomena, inculcates a rational and conservative practice; and while by its adoption we substitute a simple, intelligible, and effective etiology, for one complex, contradictory, and inadequate, we get rid of much of the fanciful theory and unfounded reasoning which have so long bewildered and disgraced our profession.

*Philadelphia, June,* 1854.

